# RETRACTED: Ahmad et al. Quantification and Evaluations of Catechin Hydrate Polymeric Nanoparticles Used in Brain Targeting for the Treatment of Epilepsy. *Pharmaceutics* 2020, *12*, 203

**DOI:** 10.3390/pharmaceutics17060686

**Published:** 2025-05-23

**Authors:** Niyaz Ahmad, Rizwan Ahmad, Ridha Abdullah Alrasheed, Hassan Mohammed Ali Almatar, Abdullah Sami Al-Ramadan, Mohd Amir, Md Sarafroz

**Affiliations:** 1 Department of Pharmaceutics, College of Clinical Pharmacy, Imam Abdulrahman Bin Faisal University, Dammam 314441, Saudi Arabia; 2160003774@iau.edu.sa (R.A.A.); 2150004453@iau.edu.sa (H.M.A.A.); 2160002963@iau.edu.sa (A.S.A.-R.); 2 Department of Pharmaceutical Chemistry, College of Clinical Pharmacy, Imam Abdulrahman Bin Faisal University, Dammam 314441, Saudi Arabia; mskausar@iau.edu.sa; 3 Department of Natural Products and Alternative Medicine, College of Clinical Pharmacy, Imam Abdulrahman Bin Faisal University, Dammam 314441, Saudi Arabia; rareiyadh@iau.edu.sa (R.A.); matahmad@iau.edu.sa (M.A.)

The journal retracts the article “Quantification and Evaluations of Catechin Hydrate Polymeric Nanoparticles Used in Brain Targeting for the Treatment of Epilepsy” [1] cited above.

Following publication, concerns were brought to the attention of the Editorial Office regarding a high level of similarity between the images and graphs presented in this publication [1] and nine other articles [2,3,4,5,6,7,8,9,10], produced by a similar authorship group.

Adhering to our standard procedure, an investigation was conducted by the Editorial Office and Editorial Board that confirmed a high level of similarity between Figure 5A in [1] and Figure 3A in [2], and between Figure 8 in [1] and Figure 7A, Figure 2A, Figure 5A, Figure 5A, Figure 6A, Figure 5A, Figure 6, and Figure 5A in [3,4,5,6,7,8,9,10]. While the authors cooperated during the investigation, they were unable to satisfactorily explain the overlap, nor provide appropriate raw material for Editorial Board evaluation. Consequently, the Editorial Board has lost confidence in the reliability of the findings and decided to retract this publication [1], as per MDPI’s retraction policy (https://www.mdpi.com/ethics#_bookmark30).

This retraction was approved by the Editor-in-Chief of the journal *Pharmaceutics.*

The authors did not provide a comment on this decision.
